# Control of multiciliogenesis by miR-34/449 in the male reproductive tract through enforcing cell cycle exit

**DOI:** 10.1242/jcs.253450

**Published:** 2021-05-11

**Authors:** Yu-Jie Wu, Yue Liu, Yan-Qin Hu, Li Wang, Fu-Rong Bai, Chen Xu, Jing-Wen Wu

**Affiliations:** 1 Department of Histoembryology, Genetics and Developmental Biology, Shanghai Jiao Tong University School of Medicine, Shanghai 200025, China; 2 Shanghai Key Laboratory of Reproductive Medicine, Shanghai 200025, China

**Keywords:** Motile cilia, Multiciliogenesis, Male reproductive tract, miR-34/449, Cell cycle

## Abstract

Multiciliated cells (MCCs) are terminally differentiated postmitotic cells that possess hundreds of motile cilia on their apical surface. Defects in cilia formation are associated with ciliopathies that affect many organs. In this study, we tested the role and mechanism of the miR-34/449 family in the regulation of multiciliogenesis in EDs using an *miR-34b/c−/−*; *miR-449−/−* double knockout (dKO) mouse model. MiR-34b/c and miR-449 depletion led to a reduced number of MCCs and abnormal cilia structure in the EDs starting from postnatal day (P)14. However, abnormal MCC differentiation in the dKO EDs could be observed as early as P7. RNA-seq analyses revealed that the aberrant development of MCCs in the EDs of dKO mice was associated with the upregulation of genes involved in cell cycle control. Using a cyclin-dependent kinase inhibitor to force cell cycle exit promoted MCC differentiation, and partially rescued the defective multiciliogenesis in the EDs of dKO mice. Taken together, our results suggest that miR-34b/c and miR-449 play an essential role in multiciliogenesis in EDs by regulating cell cycle exit.

## Abstract (precis)

**Summary:** Mutagenic, expression and histological analyses reveal an essential role for miR-34b/c and miR-449 in multiciliogenesis in efferent ductules of the male reproductive tract by regulating cell cycle exit.

## INTRODUCTION

Cilia are finger-like cellular projections composed of microtubules. There are two types of cilia: non-motile or primary cilia, which might mediate signaling perception; and motile cilia, which are required for cell motility and extracellular fluid movement (Youn and Han, 2018; Spassky and Meunier, 2017). Multiciliated cells (MCCs), characterized by the presence of multiple motile cilia at their apical surface, have been described in many vertebrates (Brooks and Wallingford, 2014). In vertebrates, MCCs line the luminal surface of some organs, including the trachea, cerebral ventricle, oviduct and efferent ductules (EDs) (Fliegauf et al., 2007; Satir and Christensen, 2008). The beating of motile cilia allows the removal of inhaled particles and pathogens from the respiratory tract, the circulation of the cerebrospinal fluid, the transportation of embryos along the oviduct, and the suspension of spermatozoa in EDs (Satir and Christensen, 2008; Yuan et al., 2019).

MicroRNAs (miRNAs) are small noncoding RNAs that regulate diverse cellular processes through binding to the 3′ untranslated regions (UTRs) of their target mRNAs, using their seed sequences, thereby functioning as post-transcriptional regulators by affecting the stability and translational efficiency of mRNA. Many miRNAs contain the same seed sequences and thus belong to a functionally related miRNA family (Bartel, 2004, 2009). The miRNA-34/449 family includes miR-34a, miR-34b, miR-34c, miR-449a, miR-449b and miR-449c. These six miRNAs have the same seed sequence; therefore, they can regulate the same mRNA targets, thus having redundant functions (Bao et al., 2012). Ablation of individual members of the miR-34/449 family in mice resulted in no developmental phenotype (Choi et al., 2011; Bao et al., 2012; Concepcion et al., 2012). Simultaneous inactivation of the miR-34/449 family in mice, either by ablation of miR-34a, miR-34/b/c and miR-449a/b/c (miR-tKO) (Song et al., 2014; Otto et al., 2017), or by ablation of miR-34b/c and miR-449a/b/c (miR-dKO) (Comazzetto et al., 2014; Wu et al., 2014), inhibited the formation of motile cilia in multiciliated epithelia. The miR-dKO mice displayed partial perinatal lethality, growth retardation, brain abnormalities, respiratory tract obstructions and infertility. Previous studies of the miR-dKO mice showed that male infertility might result from spermatogenic disruption in the testis (Comazzetto et al., 2014; Wu et al., 2014). However, recent research showed that a ciliogenesis defect in the EDs compromises the movement of sperm from the testis into the epididymis, and likely contributes to the infertility of miR-tKO males (Otto et al., 2017). Recently, we showed that loss of ED motile cilia in double knockout (dKO) mice causes sperm aggregation and luminal obstruction, which in turn, induces back-pressure atrophy of the testis and ultimately male infertility. In addition, we also found that unlike the wave-like coordinated beat of motile cilia in one direction in the respiratory tract and oviduct, the ED cilia display a whip-like beat in different directions, generating luminal turbulence, which maintains non-motile spermatozoa in suspension within the lumen (Yuan et al., 2019). The unique movement of the ED cilia suggests unique regulation of multiciliogenesis in this portion of the male reproductive tract. In this study, we tested the role and mechanism of the miR-34/449 family in the regulation of multiciliogenesis in EDs using *miR-34b/c^−/−^*; *miR-449^−/−^* miR-dKO mice.

## RESULTS

### Ablation of miR-34b/c and miR-449a/b/c caused sperm aggregation in efferent ductules

**Fig. 1.**
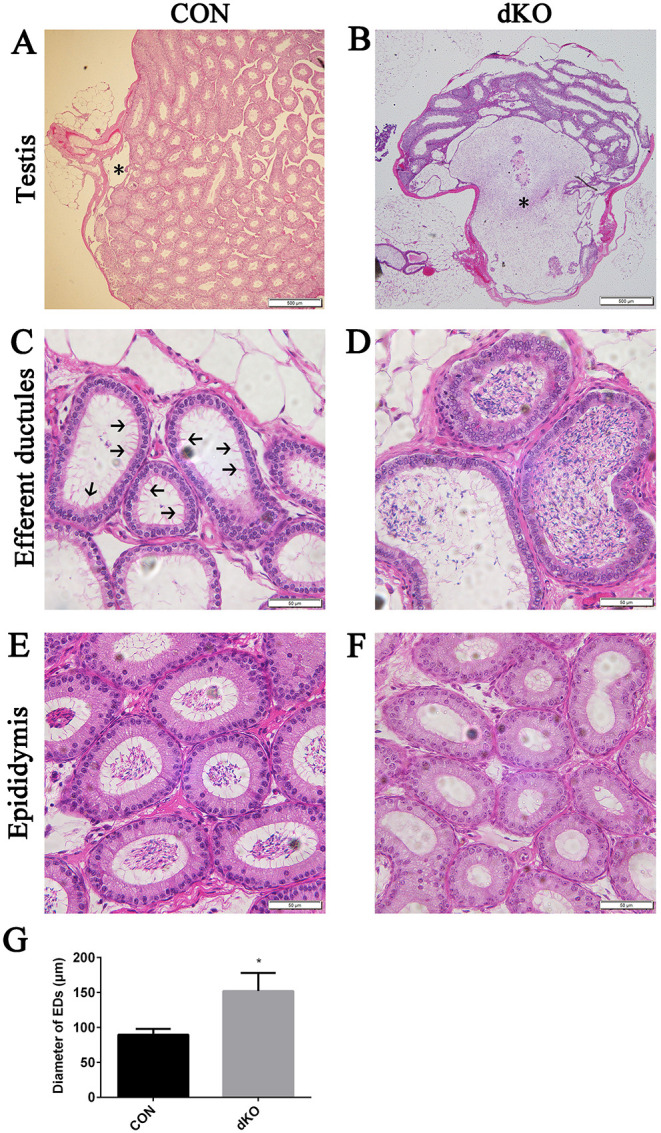
**Dilated rete testis, atrophy of seminiferous tubules and sperm aggregation in EDs in adult male miR-dKO mice.** (A,B) H&E-stained paraffin sections of the testis of miR-dKO mice showing a dilated rete testis (asterisks) and seminiferous tubule atrophy. (C,D) H&E-stained paraffin sections showing that the EDs are dilated and contain many spermatozoa in miR-dKO males. Arrows indicate the cilia in CON EDs. (E,F) H&E-stained paraffin sections showing that although spermatozoa are present in the epididymis of CON males, no spermatozoa are observed in the epididymis of dKO males. (G) Quantification of the diameter of EDs between CON and miR-dKO mice. Data are mean±s.d. (*n*=3). *P<0.05 (unpaired, two tailed Student's *t*-test). Scale bars: 500 μm (A,B); 50 μm (C-F).

We generated *miR-34b/c^−/−^*; *miR-449a/b/c^−/−^* mice (here called miR-dKO mice) as described previously (Wu et al., 2014). We found that the EDs, a set of tiny tubules that connect the rete testis to the caput epididymis, were dilated and contained many spermatozoa in miR-dKO adult males compared with control (CON) males (Fig. 1C,D,G), suggesting a physical block in the tubules preventing the transition of spermatozoa. This obstruction induced back-pressure to the rete testis and seminiferous tubules, thus causing dilated rete testis and atrophy of seminiferous tubules in male miR-dKO mice compared with CON mice (Fig. 1A,B). Although spermatozoa were present in the epididymis of CON males (Fig. 1E), no spermatozoa were observed in the epididymis of dKO males (Fig. 1F), implying that the passage of sperm from the EDs into the epididymis was compromised. We have demonstrated that multiciliogenic failure causes sperm aggregation and luminal obstruction in the EDs, which in turn, induces back-pressure dilation of rete testis and atrophy of seminiferous tubules in adult miR-dKO males. However, when does the compromised multiciliogenesis occur? To address this question, we examined the multiciliogenesis in developing EDs in miR-dKO mice and their littermate controls.

### Defection of multiciliogenesis in developing efferent ductules in miR-dKO mice

**Fig. 2.**
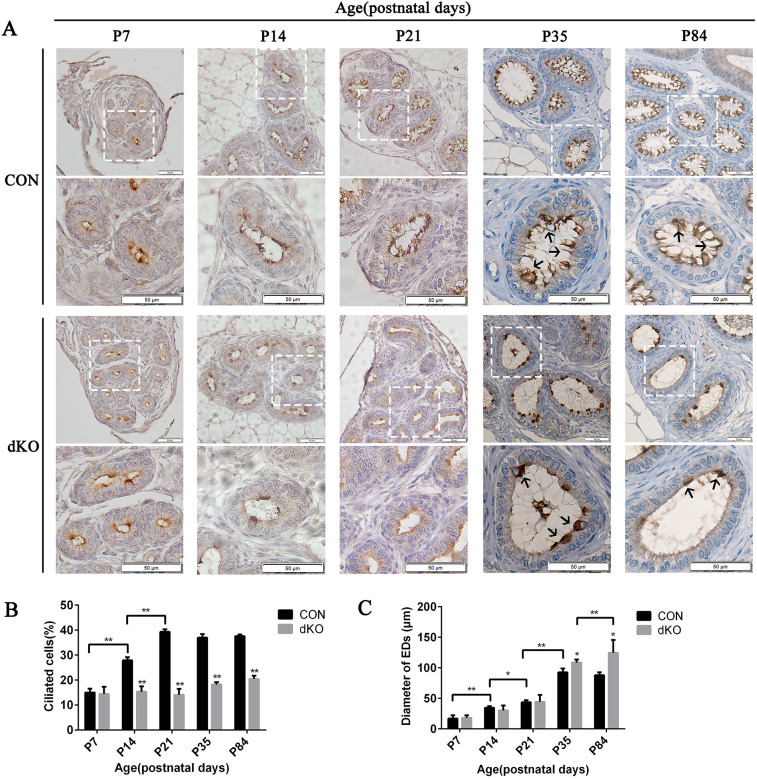
**Ciliary defects in developing EDs in miR-dKO mice.** (A) Immunohistochemical staining of Ac-α-tubulin of developing EDs showing the reduced number of MCCs (brown) and abnormal cilial structure in dKO males. Scale bars: 50 μm. The images in the lower panels of CON or dKO show the digitally amplified subfields (dashed line framed) of those in the upper panels. Black arrows show the ciliated cells. (B) Quantification of ciliated cells in EDs between CON and miR-dKO mice. (C) Quantification of the diameter of EDs between CON and miR-dKO mice. Data are mean±s.d. (*n*=3). *P<0.05, **P<0.01 (unpaired, two tailed Student's *t*-test).

We examined multiciliogenesis in developing EDs starting from postnatal day (P)7 to P84. Immunostaining for acetylated α-tubulin (Acy-α-tubulin), a marker of motile cilia, showed that the ratio of ciliated cells to total epithelial cells was not significantly different between the EDs in P7 control (CON) and dKO males [15.02±1.60% (CON) versus 14.44±2.91% (miR-dKO), *n*=3, *P*>0.05; note, all results in main text are given as mean±s.d.]. At P14, the ratio of ciliated cells increased significantly in the CON EDs, but there was no significant increase in EDs in the miR-dKO group [27.91±1.24% (CON) versus 15.31±2.20% (dKO), *n*=3, *P<*0.01]. The results indicated that the compromised multiciliogenesis starts by P14. From P21 to P84, the ratio of ciliated cells was fixed in CON EDs (at ∼32-39%), but there was still no significant increase in the miR-dKO EDs (Fig. 2A,B). Interestingly, even though there were a few Acy-α-tubulin positive ‘MCCs’ in the dKO EDs, the ‘cilia’ did not protrude into the lumen (Fig. 2A). By measuring the diameter of the developing EDs from P7 to P84, we found that the dilation of EDs in dKO males occurred by P35, and increased with age [P35, 92.57±6.300 μm (CON) versus 108.6±5.183 μm (dKO), *P*<0.05; P84, 88.09±4.421 μm (CON) versus 127.4±20.96 μm (dKO), *P*<0.05]. Interestingly, in CON males, the diameter of the EDs increased with age and peaked at P35, with no more increase up to P84. However, in dKO males, the diameter of EDs still increased from P35 to P84 (Fig. 2C). Taken together, our data demonstrate that the reduced number of MCCs and aberrant structure of MCCs occurred by P14 and the dilation of EDs in dKO males occurred by P35, and increased with age.

### miR-dKO mice displayed aberrant cell fate specification

**Fig. 3.**
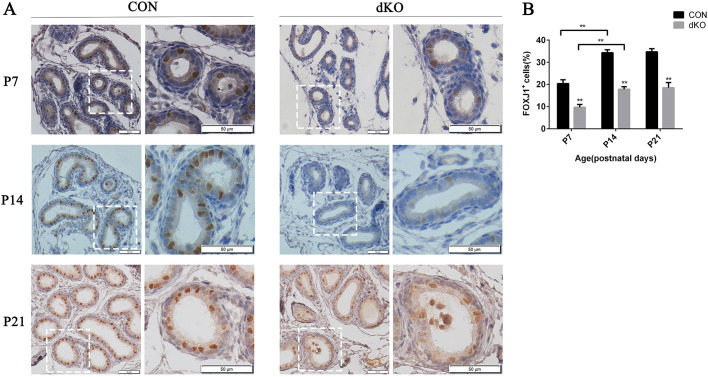
**Aberrant cell fate specification in ciliated cells in the ED epithelium of miR-dKO mice.** (A) Immunohistochemical staining for FOXJ1 showing a reduced number of FOXJ1^+^ cells (brown) in dKO males. Scale bars: 50 μm. The images in the right panels of CON or dKO show the digitally amplified subfields (dashed line framed) of those in the left panels. (B) Quantification of FOXJ1^+^ cells between CON and miR-dKO mice. Data are mean±s.d. (*n*=3). *P<0.05, **P<0.01 (unpaired, two tailed Student's *t*-test).

We found that according to the number of Acy-α-tubulin^+^ cells, there was no visible change between the miR-dKO and control males in EDs at P7. What was the status of cell fate specification of multiciliated progenitors at this time? Forkhead box J1 (FOXJ1) is an essential transcription factor for multiciliated cell differentiation (Chen et al., 1998), and has been used as a marker for multiciliated progenitor cells. We detected the FOXJ1 protein expression in the ED epithelium of miR-dKO and CON mice from P7 to P21. We found that the number of FOXJ1^+^ cells in dKO EDs was much lower than that in the controls between P7 and P21 (Fig. 3). In addition, we found that the number of FOXJ1^+^ cells increased significantly between P7 and P14 in both the dKO and CON mice. However, between P14 and P21, there was no significant increase in FOXJ1^+^ cells in either the dKO or CON mice. These results indicated that acquisition of the MCC fate might be impaired in miR-dKO mice as early as P7, despite dKO and CON mice containing a similar number of MCCs (Fig. 2).

### Ablation of miR-34b/c and miR-449a/b/c caused upregulation of cell cycle-related genes in the epithelia of efferent ductules

**Fig. 4.**
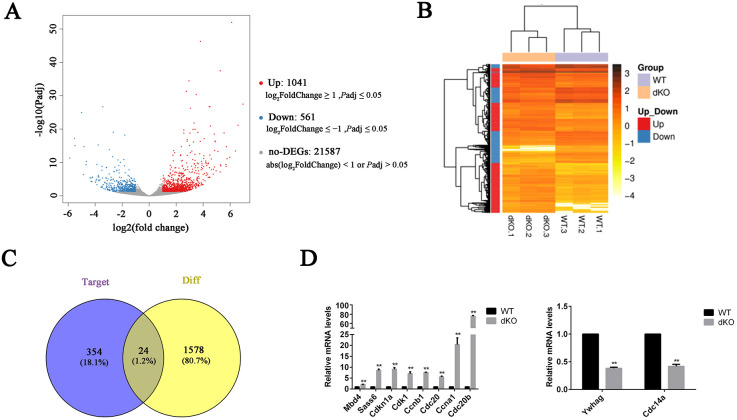
**Analysis of ED transcriptomics in wild-type and dKO mice.** (A) Volcano diagram of DEGs. (B) Heatmap of DEGs. (C) Venn diagram based on conjoint analysis between DEGs and target genes. (D) RT-qPCR validation of gene expression changes for selected cell cycle genes in the ED epithelia from miR-dKO compared with wild-type males at P21. Data are mean±s.d. (*n*=3), normalized to β-actin transcript levels. *P<0.05, **P<0.01 (unpaired, two tailed Student's *t*-test).

To further understand the molecular basis of the defect in multiciliogenesis in miR-dKO mice, we analyzed gene expression changes by performing RNA-seq using miR-dKO and wild-type EDs. We chose mice at the age of P21 for analyses because we found that multiciliogenesis in EDs was complete at this time, as shown in Fig. 2. In addition, P21 coincides with the onset of spermiogenesis, during which no sperm enter into the EDs (Bellve et al., 1977). We obtained 1602 differentially expressed genes (DEGs), of which 1041 were upregulated in miR-dKO mice and 561 were downregulated (Fig. 4A,B). We then conducted gene ontology (GO) analyses on the DEGs (Fig. S1A). Our results showed that these DEGs participated mainly in the mitotic cell cycle and centriole assembly. Most of these dysregulated genes were upregulated in dKO EDs (Table S1). Kyoto Encyclopedia of Genes and Genomes (KEGG) analyses (Fig. S1B) identified that 15 out of 1602 genes participated in cell cycle process, and 12 out of 1602 participated in the p53 signaling pathway, most of which were upregulated (Table S2). Through bioinformatic analyses, we identified 378 genes that could be targeted by miR-34/449. Only 24 out of 378 target genes were identified among the DEGs dysregulated between dKO and wild-type EDs, including 16 that were upregulated and eight that were downregulated (Fig. 4,C). Based on the above analyses, we selected ten genes (including three miR-34/449 target genes) related to the cell cycle process and verified their expression levels using quantitative real-time reverse transcription PCR (RT-qPCR) (Fig. 4D). The expression levels of *Mbd4*, *Sass6*, *Cdkn1a*, *Cdk1*, *Ccnb1* (Cyclin B1), *Ccna1*, *Cdc20* and *Cdc20b* were upregulated, whereas *Ywhag* and *Cdc14a* were downregulated in miR-dKO EDs, which was consistent with the RNA-seq results.

**Fig. 5.**
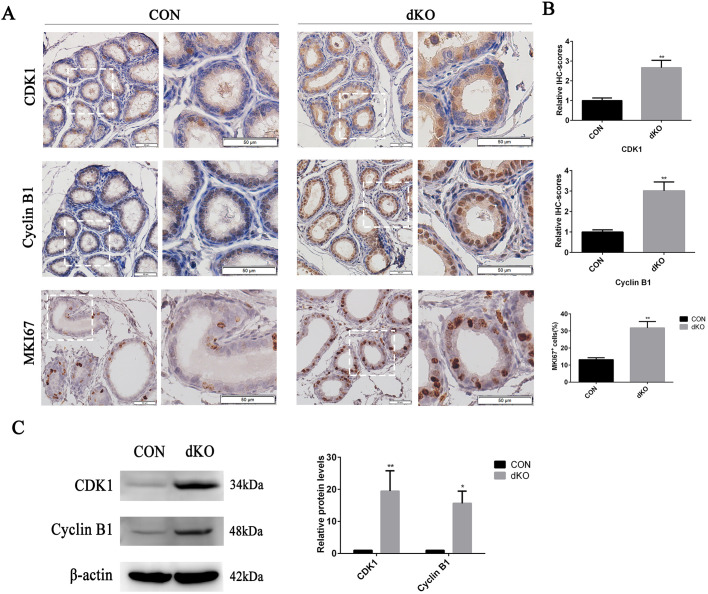
**Ablation of miR-34b/c and miR-449 causes upregulation of cell cycle-related genes in the epithelia of EDs.** (A) Immunohistochemical staining for CDK1 (upper panel), Cyclin B1 (middle panel) and MKI67 (lower panel) showing that the increased expression levels of CDK1^+^, Cyclin B1^+^ and MKI67^+^ cells (brown) in the efferent epithelia of dKO males. The images in the right panels of CON or dKO show the digitally amplified subfields (dashed line framed) of those in the left panels. Scale bars: 50 μm. (B) Quantification of the expression levels of CDK1^+^, Cyclin B1^+^ and MKI67^+^ cells in CON and miR-dKO mice. (C) Western blotting analysis of CDK1 and Cyclin B1 of the EDs from CON and dKO males at P21 (left) with the corresponding average grey levels (right). Data are mean±s.d. (*n*=3). *P<0.05, **P<0.01 (unpaired, two tailed Student's *t*-test).

We then examined the protein levels of Cyclin B1 and cyclin-dependent kinase 1 (CDK1) in miR-dKO and CON EDs using immunohistochemistry and western blotting. The results showed that both CDK1 and Cyclin B1 levels were indeed upregulated in miR-dKO EDs (Fig. 5A,B, upper two panels, Fig. 5C). Did the increased expression of cell cycle-related genes lead to abnormal proliferation of epithelial cells in miR-dKO EDs? We detected the expression of MKI67, a cell proliferation marker, in the epithelium of EDs of P21 miR-dKO mice. We found that the fraction of proliferating cells was significantly elevated in the ED epithelia of dKO mice, as revealed by MKI67 staining (Fig. 5A,B, the lowest panel). Taken together, these results suggested that ablation of miR-34b/c and miR-449a/b/c led to elevated expression of key cell cycle genes in the ED epithelium, which maintained epithelial cells in a proliferative state by preventing their exit from the cell cycle. Consequently, multiciliogenesis failed in the ED epithelium in miR-dKO mice.

### Inhibition of cell cycle progress could partially rescue multiciliogenic defects in the epithelia of EDs from miR-dKO mice *in vivo*

**Fig. 6.**
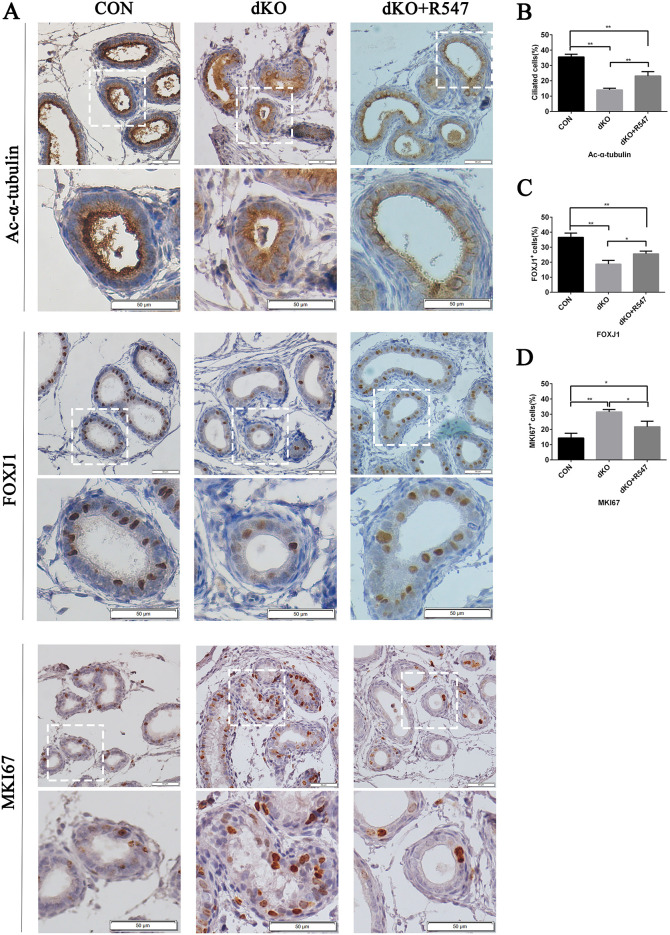
**Inhibition of cell cycle progress using a cyclin-dependent kinase inhibitor (R547) promotes MCC differentiation in ED epithelia of miR-dKO mice *in vivo*.** (A) Histological sections of ED epithelia from CON and dKO males treated with either vehicle or R547 (2 mg/kg) for 2 weeks starting from P7. The top two panels were stained for Ac-α-tubulin, the middle two panels were stained for FOXJ1, and the bottom two panels were stained for MKI67. In all sections, brown staining reflects positive immunohistochemical staining for that protein. Scale bars: 50 μm. The images in the lower panels of CON or dKO or dKO plus R547 show the digitally amplified subfields (dashed line framed) of those in the upper panels. (B) Quantification of ciliated cells (Ac-α-tubulin^+^ cells) in EDs. (C) Quantification of FOXJ1^+^ cells. (D) Quantification of MKI67^+^ cells. Data are mean±s.d. (*n*=3) *P<0.05, **P<0.01 (one-way ANOVA).

**Fig. 7.**
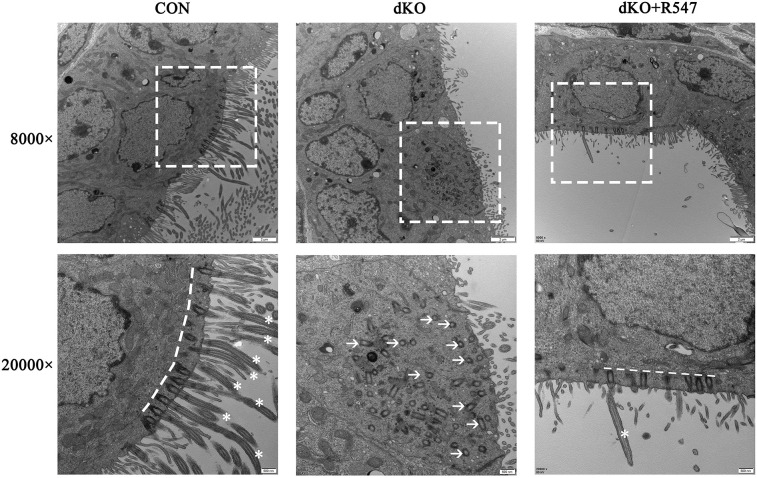
**Inhibition of cell cycle progress using a cyclin-dependent kinase inhibitor, R547, reorganizes the disorganized centrioles in ED epithelia of miR-dKO mice.** Transmission electron microscopic images of the ED epithelia from CON and dKO males treated with solvent or R547 for 2 weeks, and dKO males treated with R547 for 2 weeks from P7. In miR-dKO EDs, fewer ciliated cells were observed. In addition, multiple disorganized centrioles (white arrows) were seen in the apical cytoplasm. After R547 treatment for 2 weeks, the organized centrioles could be seen along the apical cytoplasm (white line). However, fully developed cilia were still rare. CON EDs displayed fully developed cilia (asterisks) and well organized basal bodies along the apical cytoplasm of cilia cells (white line). The images in the lower panel are the higher magnifications of those in the upper panel.

We reasoned that the failure of multiciliated cells in miR-dKO EDs resulted from failure to exit the cell cycle caused by upregulation of cell cycle regulators. To test whether forced cell cycle arrest could rescue multiciliogenic defects in miR-dKO EDs, we treated P7 miR-dKO males with R547, a CDK1/2/4 inhibitor that can inhibit the activity of CDK1/Cyclin B, CDK2/Cyclin E and CDK4/Cyclin D1 through intraperitoneal injection. Two weeks after R547 treatment, the expression of FOXJ1 was restored [36.60±2.83% (CON) versus 18.74±2.48% (dKO) versus 25.62±1.79% (dKO plus R547), *n*=3, *P*<0.05], and the hyperactive proliferation, as represented by MKI67 staining, was suppressed [14.41±3.10% (CON) versus 31.51±1.54% (dKO) versus 21.75±3.68% (dKO plus R547), *n*=3, *P*<0.05] in epithelia of EDs from miR-dKO mice. The number of Acy-α-tubulin^+^ cells was increased [35.59±1.72% (CON) versus 14.11±1.04% (dKO) versus 23.15±2.87% (dKO plus R547), *n*=3, *P*<0.01]. However, there were still no cilia protruding into the lumen (Fig. 6). We next analyzed the morphology of the cilia of miR-dKO EDs using transmission electron microscopy (Fig. 7). In miR-dKO EDs, fewer ciliated cells were observed and multiple disorganized centrioles were seen in the apical cytoplasm. However, the control EDs displayed fully developed and well-organized basal bodies along the apical surface of the ciliated cells, with numerous long motile cilia extending into the lumen. After administration of the cell cycle inhibitor for 2 weeks in dKO males, the disorganized centrioles reorganized along the apical cytoplasm. However, fully developed cilia were still rare. Our data suggest that inhibition of cell cycle progression can partially rescue the multiciliogenic defects in ED epithelia of miR-dKO mice.

## DISCUSSION

In this study, we investigated the impact of loss of miR-34b/c and miR-449 on ED multiciliogenesis. We examined the time course of the development of ciliary abnormalities, and the differentiation of the MCCs in EDs. We found that depletion of miR-34b/c and miR-449 led to a reduced number of MCCs and an abnormal cilia structure in the EDs starting from P14. However, we observed abnormal MCC differentiation in the dKO EDs as early as P7. Using RNA-seq with EDs from P21 males, we found that cell cycle genes were dysregulated in the dKO EDs. The defects in proliferation, cell fate specification and multiciliogenesis in the dKO males were partially rescued by intraperitoneal injection of a cell cycle inhibitor. Overall, our study represents an interesting step forward in our understanding of the roles of miR-34/449 in the function of EDs.

The EDs are small tubules connecting the rete testis to the caput epididymis and function to transport spermatozoa, and reabsorb water, ions and proteins (Ilio and Hess, 1994). The ED epithelium contains principal cells and ciliated cells, as well as basal cells, which are believed to be the precursors of both principal and ciliated cells. Approximately 90% of the fluid secreted by the seminiferous epithelium is re-absorbed in the EDs by the principal cells (Hess, 2000). The cilia in the EDs do not beat in the same direction; instead, they display a whip-like beating pattern in different directions, generating luminal turbulence to maintain immotile spermatozoa in suspension within the lumen (Yuan et al., 2019). The normal structure and function of the EDs are important to maintain testicular fluid homeostasis and transportation of testicular spermatozoa to the caput epididymis for maturation, which is critical for male fertility.

Multiciliogenesis is a process through which motile cilia are formed. It involves multiple events, including: (1) cell cycle exit; (2) acquisition of the MCC identity; (3) reorganization of the apical actin network; (4) multiplication of centrioles to form basal bodies; and, finally, (5) each basal body at the base of each cilium acts as a microtubule organizing center from which an axoneme elongates (Brooks and Wallingford, 2014; Chevalier et al., 2015; Meunier and Azimzadeh, 2016). Several key regulators of multiciliogenesis have been identified to date (Thomas et al., 2010), e.g. transcriptional factors, such as geminin coiled-coil domain-containing protein 1 (GEMC1) (Arbi et al., 2016; Terré et al., 2016; Vladar and Mitchell, 2016), multiciliate differentiation and DNA synthesis associated cell cycle protein (MCIDAS) (Boon et al., 2014; Kyrousi et al., 2015; Lu et al., 2019), E2F transcription Factor (E2F)4/5 (Danielian et al., 2016), FOXJ1 (Chen et al., 1998; Gomperts et al., 2004) and regulatory factor X5 (RFX5) (Chung et al., 2012, 2014). In this study, we found that the aberrant development of MCCs in the EDs of miR-dKO mice was associated with the upregulation of cell cycle genes, including CDKs. It has been reported that an alternative cell cycle program controls MCC differentiation. For example, Vladar et al. (2018) reported that CDK2, the key regulator of cell cycle entry and centrosome and DNA duplication in the S phase, is responsible for the activation of the MCC gene expression program. Otto et al. (2017) reported that upregulation of CDKs can prevent MCCs from exiting the cell cycle, thus leading to failure of MCC differentiation and, consequently, a reduced number of MCCs. Cyclin O (encoded by *Ccno* in mice) is necessary to promote deuterosome-mediated centriole amplification and the generation of multiple motile cilia (Wallmeier et al., 2014; Núnez-Ollé et al., 2017; Terré et al., 2019). The fact that inhibition of cell cycle progress using the CDK inhibitor R547 could partially rescue the defects in multiciliogenesis of miR-dKO mice, supported our hypothesis that miR-34b/c and miR-449 control MCC differentiation by promoting cell cycle exit. Several studies have shown that miR-34/449 control MCC differentiation by successively inducing cell cycle arrest, directly repressing the Notch pathway, and promoting the reorganization of the apical actin network by acting on small GTPase pathways in human airway epithelium and frog epidermis (Marcet et al., 2011; Chevalier et al., 2015; Mercey et al., 2016). Impaired ciliogenesis caused by miR-34/449 deficiency is largely caused by aberrant maturation and docking of basal bodies through the upregulation of centriolar Coiled-Coil Protein 110 (Cp110), a centriolar protein that needs to be removed from centrioles to facilitate centriole-to-basal body transition (Song et al., 2014; Walentek et al., 2016). Otto et al. (2017) reported that increased activity of cyclin-dependent kinases is responsible for the multiciliogenesis defect in the respiratory epithelium of miR34/449^−/−^ mice. The extent of ciliation in the respiratory epithelium could be fully corrected after treatment with inhibitors of cyclin-dependent kinases, such as R547. In our study, the ciliogenesis in the dKO EDs was partially rescued by R547. How can the discrepancy between the phenotypes of our mice and those previously reported after R547 treatment be explained? (Otto et al., 2017). We speculated that the multiciliogenesis processes in different organs (brain, trachea, oviduct and EDs) have common characteristics but also have tissue specificity. This tissue specificity might explain the different rescue effect we observed. For example, a previous investigation found that *E2f4* deficiency alone disrupted multiciliogenesis in the airway epithelium; however, it did not disrupt multiciliogenesis in the EDs. Multiciliated cell development fails when E2F4 is absent in combination with heterozygosity of *E2f5* (Danielian et al., 2016) in EDs. Interestingly, we found that the cilia beat in the EDs of the male is different from that in other tissues, such as the respiratory epithelium or oviduct epithelium, which display a uniform wave-like beat toward the same direction. The cilia beat in EDs produces a whip-like motion, generating a centripetal force on the luminal fluid. Each cilia cell can beat in a different direction, often opposing the direction of the cilia cells on the opposite side of the lumen (Yuan et al., 2019).

We performed RNA-seq on EDs from P21 males, because at this age the multiciliogenesis is complete and sperm are not yet present, which permitted a clear insight into the impact of miR-34b/c and miR-449 on the multiciliogenesis. We found that the cell cycle-related genes were dysregulated markedly. Our previous RNA-seq data demonstrated that the dysregulated genes in P56 miR-dKO EDs were mostly involved in ciliogenesis, including axoneme assembly, microtubule bundle formation, cilium assembly, cilium organization and other similar functions (Yuan et al., 2019). We speculated that the observed difference was the result of performing RNA-seq at different ages. Gene expression patterns in most tissue show spatiotemporal specificity. The dilation of EDs occurred by P35 and increased with age in dKO males, which suggests different gene expression patterns between P21 and P56 dKO EDs. In addition to the upregulation of cell cycle-related genes in the miR-dKO epithelium of EDs, our RNA-seq data identified some dysregulated genes that encoded proteins that participate in microtubule formation. Moreover, although the cyclin-dependent kinase inhibitor could enforce cell cycle exit and promote MCC differentiation, the cilium assembly was not markedly improved, suggesting that other factors are needed to accomplish full multiciliogenesis in the ED epithelium, in addition to proper cell cycle exit. Thus, further investigation is needed to identify the other factors required for multiciliogenesis during ED development. In summary, our study suggested that miR-34b/c and miR-449 play essential roles in multiciliogenesis during ED development by regulating proper cell cycle exit.

## MATERIALS AND METHODS

### Animals

All animal work was performed following the protocol approved by the Institutional Animal Care and Use Committee of the Shanghai Jiao Tong University School of Medicine. Mice were housed and maintained under specific pathogen-free conditions in a temperature- and humidity-controlled animal facility in the Shanghai Jiao Tong University School of Medicine. *miR-449* and *miR-34b/c* knockout mice were generated as described previously (Wu et al., 2014). All mice used in this study were based on the C57BL/6J background. All CON mice in this study were littermates of the analyzed miR-dKO mice.

### Histology analysis

EDs were dissected under a stereoscopic microscope and fixed in Bouin's solution overnight at room temperature. The tissues were embedded in paraffin after dehydration and being made transparent. Then, 5 μm sections were cut and stained using hematoxylin and eosin (H&E) after deparaffinization and rehydration. Images were observed under a microscope (Nikon, ECLIPSE E600, Tokyo, Japan).

### Immunohistochemistry analysis

Four percent paraformaldehyde-fixed paraffin-embedded EDs sections were de-waxed and rehydrated. For antigen retrieval, the tissues were boiled in 10 mM citrate buffer (pH 6.0) for 15 min. The sections were treated with 3% H_2_O_2_ for 10 min to neutralize endogenous peroxidase activity, and then blocked in counterpart IgG for 30 min at room temperature. Then, primary antibodies recognizing acetylated-α-tubulin (1:200; T6793; Sigma-Aldrich, St. Louis, MO, USA), FOXJ1 (1:200; 14-9965; eBioscience, San Diego, CA, USA), MKI67 (1:500; 12202; Cell Signaling Technology, Danvers, MA, USA), CDK1 (1:100; ab18; Abcam, Cambridge, MA, USA), and Cyclin B1 (1:50; SAB4503501; Sigma-Aldrich) were added and incubated overnight at 4°C. On the following day, the sections were incubated with a polyclonal goat anti-mouse/rabbit IgG antibody for 15 min at room temperature. The signals were visualized using a Histostain-Plus Kit (Life Technologies, Carlsbad, CA, USA) and nuclei were counterstained with hematoxylin. Images were observed under a microscope (Nikon, ECLIPSE E600, Japan).

### Measurement of the diameter of EDs and quantification of the results of the immunohistochemistry analysis

We selected 20-30 individual cross-sections of the mid-to-distal regions of EDs from each mouse to measure the diameter of the ED and quantify the results of the immunohistochemistry analysis. The identity of the samples was blinded. Only those close-to-round EDs were chosen for diameter measurement, which used ImageJ (version 1.46r; National Institutes of Health, Bethesda, MD, USA). The diameter of an individual cross-section was equal to the average value of the maximum and minimum values measured. To analyze the proportion of ciliated cells or FOXJ1- or MKI67-positive staining cells among total cells of the ED epithelium, quantification was performed using manual counting. To analyze the nuclear protein intensity of CDK1 or Cyclin B1, quantification was performed using the immunohistochemistry (IHC)-scores and the following rules: IHC-score=Σ (percentage of cells immunostained×average intensity of staining). We divided the intensity of CDK1 or Cyclin B1 staining into four levels (negative staining, 0; weak staining, 1; moderate staining, 2; and strong staining, 3). The average IHC-score of the chosen EDs for each section was used for analysis. At least three biological replicates were performed.

### RNA-seq and bioinformatic analysis

EDs of 3-week-old mice were dissected under a stereoscopic microscope and then the tissues were stored at −80°C for subsequent detection. The RNA concentration and quality were measured using an Agilent 2100 Bioanalyzer (Agilent, Santa Clara, CA, USA). RNA-seq was performed using Illumina HiSeq sequencers (Illumina, San Diego, CA, USA) at BGI Bioscience (Beijing, China). The software SOAPnuke (BGI) was used to remove adaptor sequences and low-quality reads from the sequencing data. To identify all the transcripts, we used StringTie (Pertea et al., 2015) and Cufflinks (Trapnell et al., 2012) to assemble the sequencing reads. The differential expression analysis was performed using DEseq2 (Love et al., 2014) and PossionDis (Audic and Claverie, 1997; Salkind, 2007). The output results were imported into R as data frame for downstream analyses. The target genes of the five miRNAs were acquired using the miRanda (microrna.org) and TargetScan (targetscan.org) databases. According to GO and KEGG pathway analysis, we performed enrichment analysis on the DEGs and classified them according to function (molecular function, cellular component and biological process) and biological pathways (cellular processes, environmental information processing, genetic information processing, human diseases, metabolism and organismal systems).

### RNA isolation and RT-qPCR

Total RNAs were isolated from wild-type and miR-dKO EDs using an RNAsimple Total RNA Kit (TIANGEN, Beijing, China; DP419) according to the manufacturer's instructions. Reverse transcription reactions were executed using a PrimeScript RT reagent Kit (Takara, Dalian, China; RR036). The RNA or DNA concentration and quality were measured using a NanoDrop 2000 spectrophotometer (Thermo Fisher Scientific, Waltham, MA, USA). The RT-qPCR reaction was prepared using a TB Green Premix Ex Taq II (Takara; RR820A) and performed using an Applied Biosystems 7500 instrument (ABI, Foster City, CA, USA) under conditions of 95°C for 15 s, followed by 40 cycles of 95°C for 5 s, and 60°C for 34 s. The β-actin gene was used as the internal control. The expression level of each gene was calculated using the 2^−△△Ct^ method (Livak et al., 2001) The primers used in this study were synthesized by Sangon Biotech (Shanghai, China) and their sequences are listed in Table S3.

### Western blotting analysis

Mouse EDs were homogenized in 80 µl of radioimmunoprecipitation assay lysis buffer (Thermo Fisher Scientific; 89900) per sample containing a protease inhibitor cocktail (Roche Applied Science, Basel, Switzerland; 5892970001) on ice. The samples were then centrifuged at 14,000 ***g*** for 10 min at 4°C. The proteins in the supernatant were collected and the protein concentrations were determined using a bicinchoninic acid Protein Assay Kit (Thermo Fisher Scientific; 23,225). The proteins were separated using 10% denaturing polyacrylamide gels, and then transferred to polyvinylidene difluoride membranes (Millipore, Billerica, MA, USA) using a semi-dry transfer apparatus (Bio-Rad, Hercules, CA, USA). The membranes were blocked by protein-free rapid blocking buffer for 15 min at room temperature. The primary antibodies recognizing CDK1 (1:200; ab18; Abcam), Cyclin B1 (1:500; SAB4503501; Sigma-Aldrich) and β-actin (1:1000; ab8227; Abcam) were added and incubated overnight at 4°C. On the following day, the membranes were incubated with secondary antibodies conjugated to horseradish peroxidase (1:5000, Cell Signaling Technology) for 1 h at room temperature. Enhanced chemiluminescence (Millipore, WBKLS0100) was employed to generate the signals, which were detected by a luminescent image analyzer (ImageQuant LAS 4000 mini; GE healthcare, Chicago, IL, USA). The protein bands from the western blot were quantified using ImageJ (version 1.46r).

### Intraperitoneal injection of mice

R547 (S2688, Selleckchem, Houston, TX, USA), a potent ATP-competitive inhibitor of CDK1/2/4, was dissolved in 1% hydroxyethyl cellulose, 0.2% Tween 80 and PBS at 0.08 mg/ml according to the manufacturer's instructions. We treated mice at P7 with 2 mg/kg body weight R547 for 2 weeks daily by intraperitoneal injection. For controls, we treated mice with the R547 solvent in the same way.

### Transmission electron microscopy analysis

The EDs were fixed using 2.5% glutaraldehyde in 0.1 M phosphate buffer (pH 7.4) overnight at 4°C and postfixed in 1% OsO_4_ for 2 h at 4°C. The samples were then dehydrated through grades of alcohol and embedded in Epon 618 (TAAB Laboratories Equipment, Aldermaston, UK). Ultrathin sections (70-90 nm) were cut and stained with uranyl acetate and lead citrate, and then the ultrastructure of the cilia in the EDs epithelium was observed using a transmission electron microscope (Philips CM-120, Eindhoven, The Netherlands) at 80 kV.

### Statistical analysis

All data are presented as mean±s.d., and were analyzed using GraphPad Prism 6 (GraphPad, La Jolla, CA, USA). Unpaired, two tailed Student's *t*-tests or one-way ANOVA tests were used to analyze statistical differences. *P*<0.05 was considered as a significant difference, and *P*<0.01 was considered as a highly significant difference. Each experiment was repeated three times independently.

## Supplementary Material

Supplementary information
