# Supplementary material for: Control of multiciliogenesis by miR-34/449 in the male reproductive tract through enforcing cell cycle exit
Source: J Cell Sci. 2021 May 11;134(9):jcs253450. doi: 10.1242/jcs.253450 (PMC8182409; doi:10.1242/jcs.253450)
Supplement: Supplementary information [file joces-134-253450-s1.pdf]

Supplemental Data

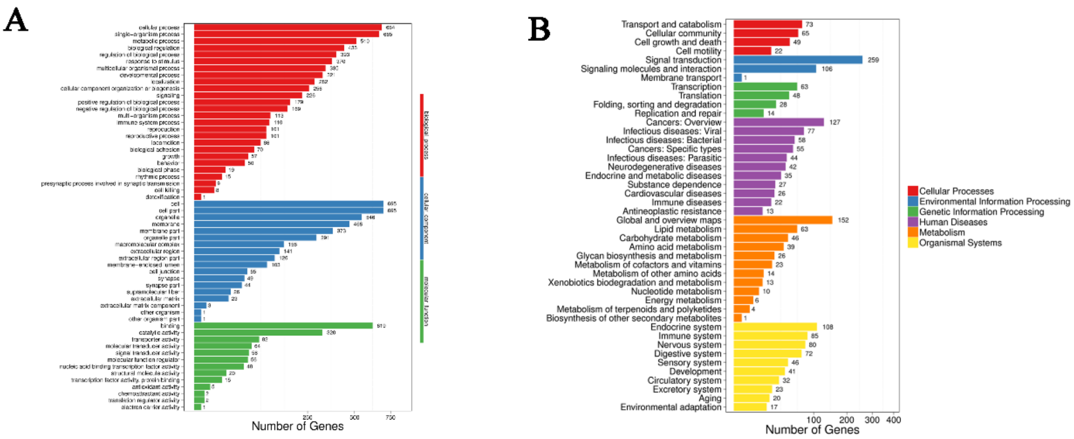

Fig. S1. Analysis of efferent ductule transcriptomics in WT and dKO mice. (A) GO analysis of DEGs. (B) KEGG pathway analysis of DEGs.

Table S1. Functional description of DEGs based on GO analysis

Description of GO terms	Genes	*P*-Value	Classification of GO terms
mitotic cell cycle	***Ywhag***, Kif20a, Pim2, Cep76, Id2, **Ccnb1**, Ptprv, Brsk2, Pak3, Spag5, Misp, Camk2a, Mcidas, Smc4, Dlgap5, Ckap2, Pcnt, Nek2, Nusap1, Cit, Rprm, Kif2c, Cdca2, Edn1, Pidd1, Birc3, Met, **Ccna1**, Trp63, E2f8, **Cdk1**, Cenpm, Rgs14, **Cdc14a**, ***Mbd4***, Cenpj, Btg4, Aurka, Ska3, Cep126, Setdb2-phf11c, E2f7, Cntrl, Nedd1, Racgap1, Smc2, Camk2b	0.00970	biological process
cell cycle process	E2f7, Nedd1, Ska3, Cep126, Sycp1, Smc2, **Ccna1**, Trp63, Cep120, Birc3, Spata22, Rgs14, Cenpm, **Cdc20**, Plk4, Pcnt, Tube1, Ckap2, Camk2a, Rprm, Cdca2, Edn1, Cit, ***Sass6***, Fancd2, Nek2, Kif20a, Pim2, Brsk2, Spag5, Ptprv, Cep135, Aurka, Setdb2-phf11c, Cenpj, ***Mbd4***, Camk2b, Cab39l, Ddias, Racgap1, Plk5, Met, Pidd1, **Cdc14a**, **Cdk1**, E2f8, Cep152, Smc4, Misp, Kif2c, Nusap1, Brdt, Hormad1, Cks2, Prkaca, Cenpe, Cep76, Id2, ***Ywhag***, Tacc3, Ccdc67, **Ccnb1**, Fbxo43	0.01451	
centriole assembly	Cenpj, Plk4, Ccdc67, Cep152, Cep135, Cep76, Ccdc78	0.01678	
cell cycle	E2f8, Ddit3, **Cdk1**, **Cdc14a**, Pidd1, Met, Rif1, Plk5, Racgap1, Ddias, Cab39l, Camk2b, ***Mbd4***, Cenpj, Setdb2-phf11c, Aurka, Cep135, **Ccnb1**,	0.04202	

Journal of Cell Science • Supplementary information

	Fbxo43, Ccdc67, Tacc3, ***Ywhag***, Id2, Cep76, Cenpe, Prkaca, Cks2, Hormad1, Brdt, Nusap1, Fignl1, Kif2c, Misp, Junb, Smc4, Cep152, **Cdc20**, Cenpm, Rgs14, Spata22, Birc3, Cep120, Trp63, **Ccna1**, Smc2, Sycp1, Sept3, Btg4, Cep126, Ska3, Cntrl, Nedd1, E2f7, Ptprv, Spag5, Pak3, Brsk2, Kif20a, Pim2, Nek2, Fancd2, ***Sass6***, Cit, Cdca2, Edn1, Rprm, Camk2a, Mcidas, Ckap2, Tube1, Dlgap5, Pcnt, Plk4		
centriole	Ccdc78, Ccdc67, Cenpj, Cep76, Plk4, Nedd1, Cep152, Cep128, Pcnt, Cep120, Cep135	0.00352	cellular component
deuterosome	Cep152, Ccdc67, Plk4, Ccdc78	0.00339	

Note: Red indicates significantly up-regulated DEGs; Blue indicates significantly down-regulated DEGs; Italic indicates target genes; Bold indicates DEGs validated subsequently.

Journal of Cell Science • Supplementary information

Table S2. Functional description of DEGs based on KEGG pathway analysis

Description of KEGG pathways	Genes	*P*-Value	Classification of KEGG pathways
p53 signaling pathway	**Cdkn1a**, Igfbp1, Shisa4, Sfn, Rprm, Perp, Pidd1, Adgrb1, **Ccnb1**, Serpinb5, **Cdk1**, Cycs	0.05425801	Cellular Processes：Cell growth and death
Cell cycle	Plk1, **Ccna1**, **Cdkn1a**, Smc1b, **Cdc20**, Dbf4, Gm5936, Sfn, ***Ywhag***, **Ccnb1**, **Cdc14a**, **Cdk1**, Gm5640, **Cdc20b**	0.1688805	

Note: Red indicates significantly up-regulated DEGs; blue indicates significantly down-regulated DEGs; Bold indicates DEGs validated subsequently.

Table S3. Primer sequences for RT-qPCR

Gene Symbol	Forward primer (5'-3')	Reverse primer (5'-3')
Mbd4	TTGGGATCGCTGCAAGGAAG	CAGTGGGCTCTTGGAGAAGG
Sass6	CTACTGGGCTTCTTCACCCTC	CTCTGGGAAAGCTTCCTGTGTA
Cdkna1	CAGAATAAAAGGTGCCACAGGC	CGTCTCCGTGACGAAGTCAA
Cdk1	ACACACGAGGTAGTGACGC	TCTGAGTCGCCGTGGAAAAG
Ccnb1	CTCCAAGCCCGATGGAAACA	TGACTGCTCTTCCTCCAGTTG
Ccna1	TTCCCAAGCAGCAGCAGG	CTAGCACGGTTCTCTGTGGG
Cdc20	CCCCAGAAGGCTACCAGAAC	GATTTCGGGGGCATCAAGGA
Cdc20b	GCACTTCCACACACAAGCTG	GCACTTCCACACACAAGCTG
Ywhag	CGATCCTCTTCAGCCCTGTG	GTTCAGCTCGGTCACGTTCT
Cdc14a	CAGGCTCGGTGATTGGGAC	AATCGATCTTTCATGAACTCGCAA
β-actin	ACTGTCGAGTCGCGTCCA	ATCCATGGCGAACTGGTGG
